# The effects of metabolic indicators and immune biomarkers on pregnancy outcomes in women with recurrent spontaneous abortion: a retrospective study

**DOI:** 10.3389/fendo.2023.1297902

**Published:** 2024-01-17

**Authors:** Jie Zhang, Zhan Song, Hui Yuan, Zhu-Hua Cai

**Affiliations:** ^1^ Department of Obstetrics and Gynecology, The Third Affiliated Hospital of Wenzhou Medical University, Wenzhou, Zhejiang, China; ^2^ Key Laboratory of Digital Technology in Medical Diagnostics of Zhejiang Province, Dian Diagnostics Group Co., Ltd., Hangzhou, Zhejiang, China

**Keywords:** recurrent spontaneous abortion, thyroid autoimmunity, immune biomarker, metabolic indicator, pregnancy outcome

## Abstract

**Background:**

The etiology of recurrent spontaneous abortion (RSA) remains elusive despite specific investigations affirming the association between RSA and thyroid autoimmunity (TAI). This study explores the immunological and metabolic profiles of RSA patients exhibiting positive thyroid antibodies and their connection with the rates of first-trimester miscarriage and live births. The aim is to provide further guidance for clinical interventions.

**Methods:**

A retrospective analysis included 478 women with RSA. Thyroid profile, thyroid peroxidase antibodies, and anti-thyroglobulin antibodies were measured in all participants. The clinical characteristics and pregnancy outcomes of RSA women were compared between thyroid autoimmunity (TAI)-positive and TAI-negative patients. Significant factors associated with adverse pregnancy outcomes and risk prediction models were explored in TAI-positive patients. Correlation analysis was used to identify specific metabolic or immune biomarkers associated with thyroid autoantibodies.

**Results:**

The prevalence of TAI was 18.6%. Compared with women without TAI, the thyroid-stimulating hormone (TSH) concentration of TAI-positive RSA was significantly higher (2.80 ± 2.98 vs 1.89 ± 1.17, p=0.006). After 28 weeks, the live birth rate of the TAI-positive group was lower than that of the TAI-negative group, with statistical significance (p<0.05). The immune biomarkers that differed between RSA women with live births and those with first-trimester miscarriages were complement C4 and interleukin-6, respectively, in TAI-negative and TAI-positive women. Then, a risk prediction model for first-trimester miscarriage was constructed for TAI-positive women with an AUC of 0.81. Finally, some factors related to thyroid peroxidase antibody (TPO-Ab) levels were explored, and it was found that TPO-Ab levels were positively correlated with free thyroxine and negatively correlated with 25 hydroxyvitamin D, interleukin-4, and fasting blood glucose in RSA patients.

**Conclusion:**

TAI-positive RSA patients have higher first-trimester miscarriage rates and a lower live birth rate, which may be related to metabolic immune shifts in TAI-positive RSA patients.

## 1 Introduction

Recurrent spontaneous abortion (RSA), defined as the loss of two or more pregnancies before 24 weeks of gestation, is a significant health issue affecting expectant parents’ physical and mental health (1). About 1-2% of women who tried to conceive had recurrent miscarriages (1). The pathogenesis of RSA is complex, and the etiology of RSA is still unclear in approximately half of cases. Common factors affecting the RSA rate include genetics, endocrine disorders, chromosomal abnormalities, autoimmune abnormalities (2). Among the numerous studies exploring etiological mechanisms, a notable connection has been established between thyroid autoimmunity (TAI) and the risk of RSA (3, 4). Clinical practice recommends that RSA patients undergo routine thyroid function testing, including triiodothyronine (T3), thyroxine (T4), and free triiodothyronine (FT3), free thyroxine (FT4), thyroglobulin antibody (TG-Ab), and anti-thyroid peroxidase antibody (TPO-Ab) (5).

Thyroid disease in pregnant women can elevate the risk of adverse maternal and fetal outcomes, including pre-eclampsia, abortion, stillbirth, premature delivery, and fetal growth restriction (6–8). Thyroid autoimmunity (TAI) is frequently encountered in women of reproductive age and stands as the predominant cause of thyroid dysfunction. Typically, TAI manifests alongside positive TPO-Ab or TG-Ab, and to a lesser degree of anti-thyroid-stimulating hormone receptor antibody (TSHR-Ab) (9). Approximately to 18% of pregnant women test positive for either TPO-Ab or TG-Ab (10). Stagnaro Green et al. initially substantiated the correlation between pregnancy loss and thyroid antibodies through a prospective observational study (11). Subsequent research has suggested a higher risk of RSA among women with positive anti-thyroid antibodies despite having normal thyroid function (6, 12). Recent research have reported an association between the presence of TPO-Ab/TG-Ab and occurrences of thrombotic events and obstetric complications linked to placental pathology, particularly placental inflammation (6, 13). Moreover, it has been observed that TPO antibodies possess the capability to traverse the placental barrier (10). The deleterious impact of autoimmune antibodies, notably on the cellular constituents within the placental villous zone, is speculated to contribute to the onset of placental inflammation. Because this is not easily detected during pregnancy, serological testing of immune biomarkers is needed to assist in detecting these risks and enabling appropriate clinical interventions.

While an association between TPO-Ab and diminished fertility rates and early pregnancy loss exists, the precise underlying pathophysiological mechanisms remain ambiguous. In addition, there are few reports on the clinical characteristics, immune biomarkers and metabolic indicators of live births compared with miscarriages in RSA women with TAI when they became pregnant again. Hence, the primary objective of this study is to investigate the immunological and metabolic profiles of individuals diagnosed with RSA who exhibit positive thyroid antibodies. Furthermore, this research endeavors to elucidate the potential associations between these indicators and the incidence rates of first-trimester miscarriages as well as successful live births.

## 2 Materials and methods

### 2.1 Study participants

This study retrospectively analyzed the clinical data of RSA patients who went to The Third Affiliated Hospital of Wenzhou Medical University after they got pregnant again from January 2021 to December 2022—inclusion criteria: two or more consecutive pregnancy losses before 24 weeks of gestation. The exclusion criteria are: a) Ectopic pregnancy and Molar pregnancy and incomplete clinical records; b) ultrasound-confirmed Uterine malformation; c) abnormal karyotype; d) hormone or metabolic disorder other than thyroid dysfunction; e) acquired thrombotic tendency; f) known clinical autoimmune diseases; g) chronic diseases; h) severe reproductive system infection. The first-trimester miscarriage (FTM) was defined as the pregnancy loss before 12 weeks, live birth (LB) was defined as the successful delivery of one or more babies after 28 weeks, and the late-trimester miscarriage (LTM) was defined as the pregnancy loss after the first 14 weeks of pregnancy, but before 24 weeks. All participants signed informed consent, which was reviewed and approved by the Ethics Committee of The Third Affiliated Hospital of Wenzhou Medical University.

### 2.2 Anti-thyroid antibodies assay

Anti-thyroid antibodies, including thyroid peroxidase antibody (TPO-Ab) and thyroglobulin antibody (TG-Ab), were analyzed using a chemiluminescence assay (Siemens IM1600). According to the status of TPO-Ab and TG-Ab, the study subjects were divided into TAI-positive groups and TAI-negative groups. Antibiotic positivity is when the anti-TG antibody levels are >4.5 IU/ml or anti-TPO antibody levels are >60 IU/ml.

### 2.3 Clinical features classification

RSA patients’ age, height, weight, blood pressure, and reproductive history were recorded at the time of hospital admission. Then, these women were divided into three groups based on calculated body mass index (BMI):<18.5 kg/m^2^, 18.5-24 kg/m^2^, and ≥24 kg/m^2^. In addition, we classified the participants into three groups in two categories of their blood pressure values (systolic blood pressure:< 110 mm/Hg, 110-140 mm/Hg, and ≥140 mm/Hg; diastolic blood pressure:<60 mm/Hg, 60-90 mm/Hg, >90 mm/Hg). For reproductive history, the women were categorized into three groups based on previous incidences of miscarriages: 2, 3, and ≥ 4, while previous miscarriage and previous term birth were classified into 0 and ≥1.

### 2.4 Metabolic indicators measurements

Thyroid stimulant hormone (TSH), free thyroxine (T4), and total triiodothyronine(T3) were detected using chemiluminescence analysis (Siemens IM1600). Fasting blood glucose (FBS), cholesterol (CHO), triglyceride (TG), high-density lipoprotein cholesterol (HDL-c), low-density lipoprotein cholesterol (LDL-c), total bilirubin (TB), and homocysteine (HCY) levels were detected by full-automatic biochemical analyzer (Siemens CH930). When measuring the concentration of 1,25-(OH) D3 in the serum of patients, we used the liquid chromatography-tandem mass spectrometry method (Siemens IM1600, Shanghai, China).

### 2.5 Immune biomarker measurements

The automatic biochemical analyzer (Siemens CH930, Shanghai, China) was used to measure the complement C3, complement C4, and complement C1q (rate turbidimetry). Flow cytometry (BD FACSCanto II, Shanghai, China) was used to determine the content of natural killer (NK) cells and total B cells in the lymphocyte subsets. The levels of plasma cytokines interleukin-2(IL-2), IL-4, IL-6, IL-10, tumor necrosis factor-alpha (TNF-α), and interferon-gamma (IFN-γ) were detected by flow fluorometry (BD FACSCanto II, Shanghai, China).

### 2.6 Statistical analysis

All statistical analyses were performed with SPSS Statistics version 19 (IBM Corp., Armonk, NY, USA) and R program version 4.2.3. Live birth rates were compared between the TAI- group and the TAI+ group stratified by clinical features. Levels of metabolic indicators and immune biomarkers were compared between TAI− and TAI+ groups stratified by pregnancy outcome. The continuous variables of clinical characteristics of study populations were presented as mean (standard) deviation or median (interquartile range). Categorical variables were presented as percentages and numbers. Continuous data were compared between the two groups by Welch two sample t-test or Wilcoxon rank sum test as appropriate, whereas categorical variables were compared using the Chi-square test or Fisher exact test as appropriate. P<0.05 was considered statistically significant. A multivariable logistic regression model was built to predict the risk of early miscarriage in RSA women with TAI. The factors included in the model were first ranked using random forest regression and then selected through model optimization (14).

## 3 Results

During the period for this retrospective cohort study, a total of 795 women with RSA had been admitted to our hospital. We included 478 pregnancies for analysis based on the inclusion and exclusion criteria described.

### 3.1 Characteristics of participants

Of the 478 patients, 389 women were without TAI, and 89 women had TAI. Women with TAI or without TAI had similar ages, height, weight, BMI, blood pressure, and reproductive history (**Table 1**). The level of TSH was statistically higher in the TAI-positive group than in the TAI-negative group. The two groups had no differences in three pregnancy outcomes (LB, FTM, and LTM). At the same time, there was borderline statistical significance in the two pregnancy outcomes (LB and FTM), as shown in **Supplementary Table 1**.

**Table 1:** Clinical features of recurrent miscarriage in women with and without TAI (variables are presented as mean (SD) or number (percentage).

Variables	TAI ^–^ (N = 389) *^1^*	TAI ^+^ (N = 89) *^1^*	p-value*^2^*
**Age (years)**	30.0 (27.0, 33.0)	30.0 (27.0, 33.0)	0.553
**Height (cm)**	160.0 (157.0, 163.0)	160.0 (157.0, 163.0)	0.147
**Weight (kg)**	54 (50, 59)	53 (48, 60)	0.845
**BMI** (**kg/m^2^)**	21.09 (19.38, 23.14)	20.83 (18.92, 23.63)	0.437
**TSH (mIU/L)**	1.65 (1.16, 2.43)	2.01 (1.33, 3.32)	0.003
**Free T4 (mIU/L)**	14.46 (13.19, 16.02)	14.93 (13.75, 16.35)	0.050
**Total T3 (mIU/L)**	1.53 (1.37, 1.75)	1.51 (1.35, 1.84)	0.785
**Gestation Age (days)**	50.0 (41.0, 53.0)	46.0 (41.0, 53.0)	0.322
**Previous term birth**			0.122
0	260 (67%)	67 (75%)	
≥1	129 (33%)	22 (25%)	
**Previous miscarriages**			0.406
2	251 (65%)	57 (64%)	
3	91 (23%)	25 (28%)	
≥4	47 (12%)	7 (7.9%)	
**Pregnancy outcome**			0.112
LB	314 (80.7%)	63 (70.8%)	
FTM	62 (15.9%)	22 (24.7%)	
LTM	13 (3.34%)	4 (4.49%)	

^1^ Median (IQR); n (%).

^2^ Wilcoxon rank sum test; Fisher’s exact test.

### 3.2 Differences in the live birth rates of patients with and without TAI: stratified by clinical characteristics

For all live-birth women and first-trimester miscarriage women, the overall live-birth rate was higher in the TAI-negative group with borderline statistical significance (83.5% vs. 74.1%, p = 0.061) (**Supplementary Table 1**). When stratified by clinical characteristics, it was noticed that women aged below 35, had normal systolic blood pressure, and had full-term labor more than once achieved a higher live birth rate in the TAI- group than in the TAI+ group (**Table 2**). In addition, higher live birth rates were also observed in the TAI- group in categories of normal BMI and normal diastolic blood pressure with borderline statistical significance. Although showing no statistical significance, women in the TAI- group also yielded higher live birth rates in other categories.

**Table 2:** Comparison of live birth rates between patients with and without TAI stratified by clinical features.

Variables	TAI^-^ N = 376*^1^*	TAI^+^ N = 85*^1^*	p-value*^2^*
Advanced age (≥35 years)			
**yes**	**78% (51/65)**	**50% (8/16)**	**0.030**
no	85% (263/311)	80% (55/69)	0.323
BMI (kg/m^2^)			
<18.5	84% (43/51)	69% (11/16)	0.274
18.5–<24	83% (218/262)	71% (35/49)	0.052
≥24	84% (53/63)	85% (17/20)	>0.999
Systolic blood pressure (mm/Hg)			
< 110	80% (95/119)	81% (22/27)	0.846
**110-140**	**85% (212/248)**	**73% (40/55)**	**0.022**
>140	78% (7/9)	33% (1/3)	0.236
Diastolic blood pressure (mm/Hg)			
<60	83% (29/35)	75% (9/12)	0.674
60-90	83% (277/332)	74% (52/70)	0.071
>90	89% (8/9)	67% (2/3)	0.455
Previous term birth			
0	86% (215/251)	80% (51/64)	0.239
**≥1**	**79% (99/125)**	**57% (12/21)**	**0.028**
Previous miscarriage			
2	82% (199/242)	77% (41/53)	0.409
3	87% (76/87)	76% (19/25)	0.205
**≥4**	**83% (39/47)**	**43% (3/7)**	**0.036**

^1%^ (n/N).

^2^ Pearson’s Chi-squared test; Fisher’s exact test.

The meaning of the bold values is statistical significance (p<0.05).

### 3.3 Differences in the clinical characteristics, metabolic indicators, and immune biomarker levels of live-birth women and first-trimester miscarriage women: stratified by TAI

Since the presence or absence of TAI is an essential factor in the pregnancy outcome of RSA patients, the clinical characteristics, immune markers, and metabolic molecules of women with live births and early abortions were compared in the TAI-positive and TAI-negative groups, respectively (**Table 3**). Among those TAI-negative RSA women, early miscarriages tended to have higher C4 levels (p = 0.03) and lower fasting glucose levels (p = 0.09). Moreover, they had lower total T3 levels (p = 0.073) and higher HCY levels (p = 0.071), and TSH levels (p=0.069) with borderline statistical significance. However, among women with positive TAI, those with first-trimester miscarriage were older (p = 0.019) and had lower interleukin-6 levels (p = 0.02).

**Table 3:** Comparison of clinical features, immune biomarkers, and metabolic indicators between first-trimester miscarriage (FTM) women and live birth (LB) women, respectively, in the TAI-negative and the TAI-positive groups.

	TAI^-^			TAI^+^		
	LB (N=314)	FTM (N=62)	p.overall	LB (N=63)	FTM (N=22)	p.overall
**Age (years)**	29.0 (27.0,32.8)	31.0 (27.0,33.8)	0.268	29.5 (4.07)	32.6 (5.40)	**0.019**
**Height (cm)**	160 (157,163)	160 (158,163)	0.97	160 (5.32)	161 (3.99)	0.505
**Weight (kg)**	53.8 (49.5,58.5)	54.2 (49.8,60.0)	0.492	54.8 (48.5,62.0)	51.2 (48.2,55.0)	0.222
**BMI (kg/m^2^)**	21.0 (19.4,22.9)	21.7 (20.0,23.2)	0.38	21.3 (19.0,24.1)	20.0 (18.7,21.6)	0.176
**Systolic blood pressure**	**117 (108,125)**	**112 (107,122)**	**0.085**	114 (12.0)	117 (14.1)	0.451
**(mm/Hg)**						
**Diastolic blood pressure**	72.0 (65.0,78.0)	72.0 (65.0,78.8)	0.941	70.1 (9.79)	70.0 (10.1)	0.969
**(mm/Hg)**						
**Gestation Age (day)**	50.0 (41.0,53.0)	50.0 (39.0,53.0)	0.818	46.0 (39.5,53.0)	45.0 (43.0,53.0)	0.672
**Previous Term birth**			0.149			0.078
0	215 (68.5%)	36 (58.1%)		51 (81.0%)	13 (59.1%)	
≥1	99 (31.5%)	26 (41.9%)		12 (19.0%)	9 (40.9%)	
**Previous miscarriages**			0.54			0.167
2	199 (63.4%)	43 (69.4%)		41 (65.1%)	12 (54.5%)	
3	76 (24.2%)	11 (17.7%)		19 (30.2%)	6 (27.3%)	
≥4	39 (12.4%)	8 (12.9%)		3 (4.76%)	4 (18.2%)	
**TSH (mIU/L)**	**1.64 (1.14,2.38)**	**1.87 (1.33,2.94)**	**0.069**	2.06 (1.48,3.61)	1.82 (1.18,3.18)	0.38
**Total T3 (mIU/L)**	**1.59 (0.30)**	**1.51 (0.31)**	**0.073**	1.49 (1.34,1.85)	1.59 (1.47,1.77)	0.189
Free T4 (mIU/L)	14.7 (2.32)	14.8 (2.27)	0.89	15.1 (2.27)	15.6 (2.28)	0.401
**HCY (umol/L)**	**6.10 (5.30,6.90)**	**6.30 (5.62,7.55)**	**0.071**	6.30 (5.65,7.30)	6.40 (5.95,7.20)	0.534
VD (ng/ml)	21.1 (15.9,26.4)	22.3 (17.7,25.4)	0.671	13.8 (3.94)	14.4 (3.89)	0.526
FBG (mmol/L)	4.62 (4.23,5.20)	4.52 (4.04,5.05)	0.09	4.18 (3.84,4.66)	4.42 (3.93,4.82)	0.268
CHO (mmol/L)	3.86 (3.46,4.38)	3.93 (3.50,4.22)	0.908	4.13 (3.43,4.66)	3.54 (3.27,4.28)	0.154
TG (mmol/L)	0.90 (0.68,1.30)	0.89 (0.68,1.28)	0.845	0.90 (0.69,1.42)	0.84 (0.70,0.96)	0.32
HDL-c (mmol/L)	1.24 (1.08,1.44)	1.28 (1.11,1.40)	0.748	1.31 (1.14,1.46)	1.28 (1.19,1.39)	0.996
LDL-c (mmol/L)	2.16 (1.78,2.61)	2.21 (1.87,2.60)	0.758	2.17 (1.74,2.95)	2.04 (1.67,2.38)	0.278
Tbil (umol/L)	10.5 (7.60,14.2)	10.6 (8.55,15.1)	0.311	9.90 (6.90,14.2)	11.1 (9.33,12.1)	0.598
Dbil (umol/L)	4.20 (3.10,5.60)	4.20 (3.30,5.77)	0.858	4.10 (2.65,5.55)	4.15 (3.10,4.60)	0.972
IBil (umol/L)	6.30 (4.50,8.70)	6.75 (5.43,9.00)	0.165	6.60 (4.65,9.25)	7.20 (5.82,7.83)	0.55
Complement C3 (g/l)	0.89 (0.79,1.04)	0.93 (0.80,1.11)	0.11	0.93 (0.78,1.08)	0.84 (0.76,1.04)	0.281
**Complemen**t **C4 (g/l)**	**0.20 (0.15,0.24)**	**0.22 (0.17,0.26)**	**0.03**	0.19 (0.16,0.24)	0.16 (0.14,0.22)	0.119
Complement C1q (g/l)	171 (150,192)	176 (150,196)	0.492	163 (144,180)	167 (135,192)	0.648
NK (%)	10.0 (6.90,15.5)	10.6 (7.95,14.4)	0.912	9.40 (6.05,14.8)	8.25 (6.73,13.4)	0.591
Total B cells (%)	13.9 (10.4,16.8)	13.1 (11.2,16.9)	0.904	15.5 (5.25)	14.7 (3.56)	0.445
IL-2 (pg/ml)	0.72 (0.33,1.22)	0.64 (0.29,1.03)	0.38	0.81 (0.48,1.64)	0.97 (0.55,1.34)	0.692
IL-4 (pg/ml)	0.85 (0.44,1.44)	0.84 (0.45,1.34)	0.811	0.64 (0.22,1.06)	0.79 (0.33,1.08)	0.553
**IL-6 (pg/ml)**	2.16 (1.29,3.30)	2.05 (1.32,3.11)	0.667	**2.67 (2.08,3.95)**	**1.96 (1.57,2.55)**	**0.02**
IL-10 (pg/ml)	0.95 (0.50,1.53)	0.87 (0.48,1.35)	0.34	1.04 (0.55,1.32)	1.03 (0.55,1.51)	0.972
**IFN-γ (pg/ml)**	0.87 (0.48,1.21)	0.90 (0.62,1.44)	0.214	**0.91 (0.48,1.33)**	**1.26 (0.70,1.91)**	**0.063**
TNF-α (pg/ml)	0.73 (0.40,1.93)	0.66 (0.45,1.92)	0.68	0.94 (0.52,4.08)	0.92 (0.55,1.12)	0.489

^1^ Median (IQR); Mean(SD); n (%).

^2^ Wilcoxon rank sum test; Welch Two Sample t-test; Fisher’s exact test.

The meaning of the bold values is statistical significance (p<0.05).

### 3.4 Variables related to first-trimester miscarriage in TAI-positive patients: random forest regression and logistic regression analysis

In TAI-positive RSA patients, factors associated with FTM screened and ranked by random forest are presented in **Supplementary Figure 1**. Based on the factors sorted by random forest, logistic regression analysis was performed, and the final optimal model included seven predictors that were associated with composite adverse pregnancy outcomes: age, interleukin-6, total bilirubin, total triiodothyronine, interferon-γ, previous miscarriages, and complement C1q.

**Figure 1:**
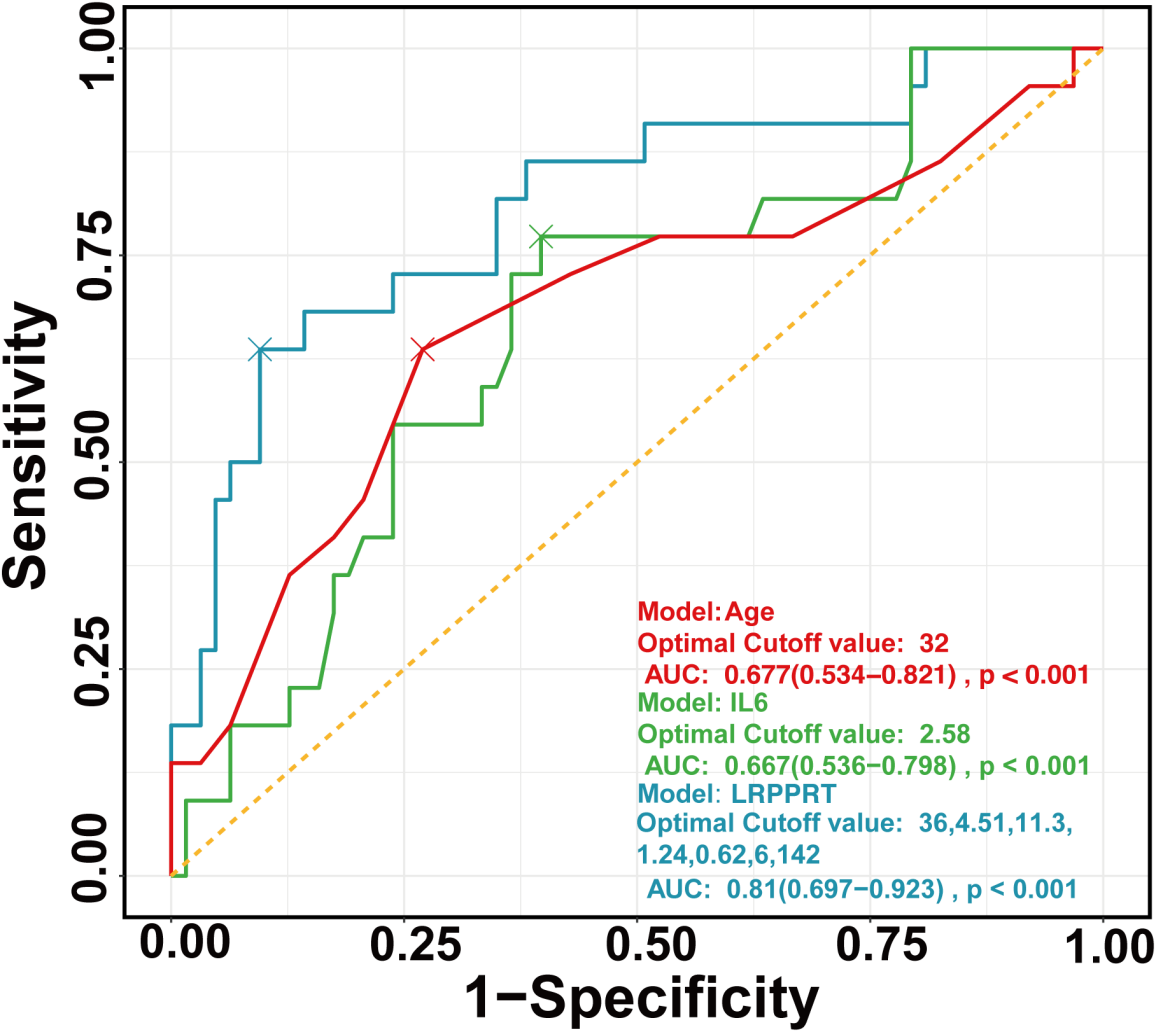
ROC curves of regression models constructed by age (red), IL-6 (green), and RF (blue), respectively. ROC, Receiver operating characteristic curve.

**Figure 2:**
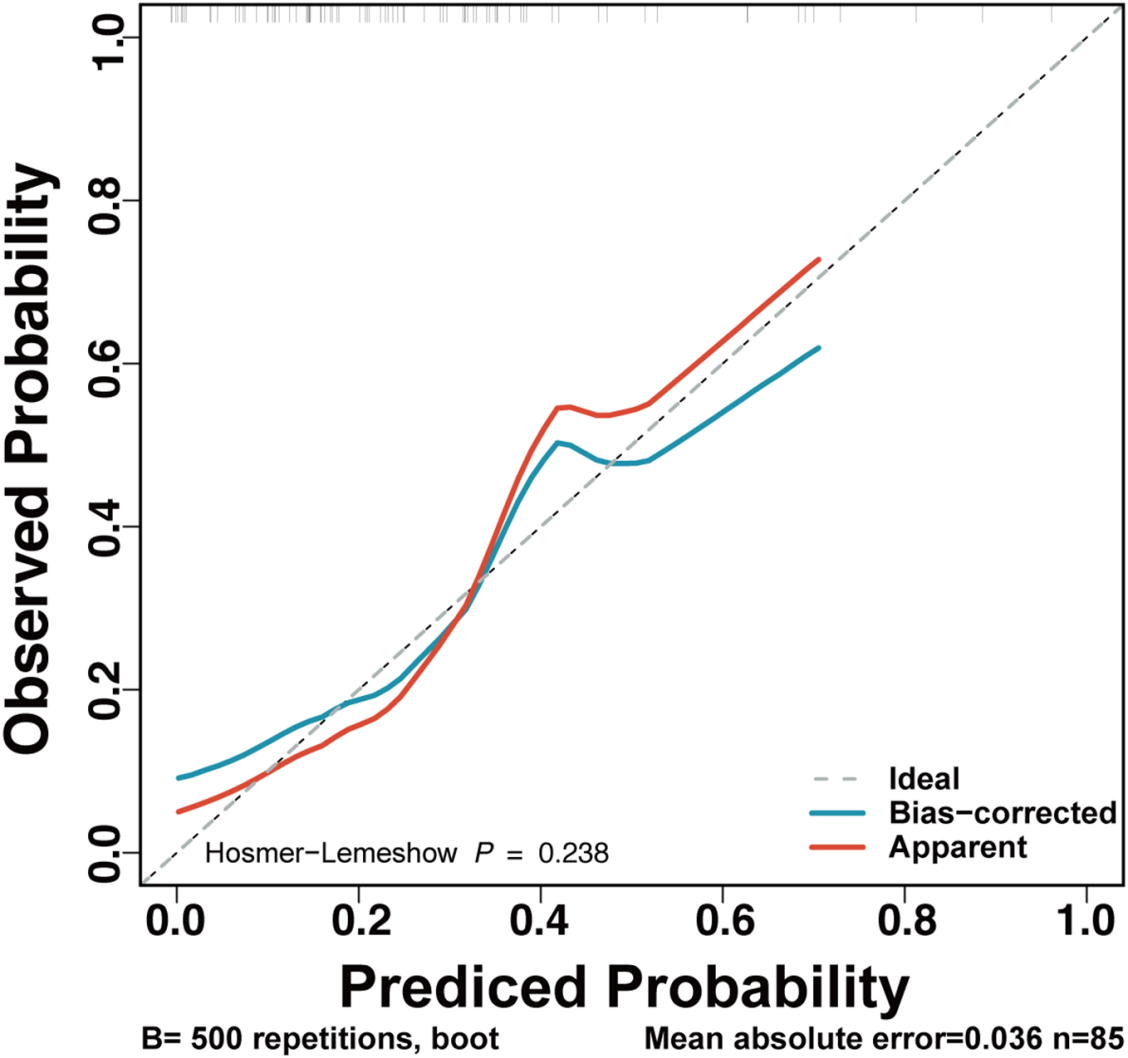
Calibration plot for the LRPPRT RSA model. This figure provides the calibration curves for the original (red) and recalibrated (blue) LRPPRT model, for the TAI-positive RSA women. Observed probabilities of mechanical ventilation (y‐axis) are plotted against predicted risks based on the RF model (x‐axis).

The final full LRPPRT (logistic regression model for predicting pregnancy outcome in RSA women with TAI) model based on random forest-screened factors is presented in **Box 1**. The final model AUC was 0.81 (95% CI 0.697 to 0.923) to discriminate between women with and without an adverse pregnancy outcome (**Figure 1**). There was a better performance of the LRPPRT logistic regression model (AUC = 0.81) when compared to age (AUC = 0.677) or IL-6 logistic regression model (AUC = 0.667) (**Figure 1**). The Hosmer-Lemeshow goodness-of-fit test shows that the LRPPRT model also had good calibration (p = 0.238) (**Figure 2**).

### 3.5 Correlation between thyroid function-related antibody levels and metabolic indicators, immune biomarkers in all RSA patients, live-birth patients, and first-trimester miscarriage patients

**Figure 3:**
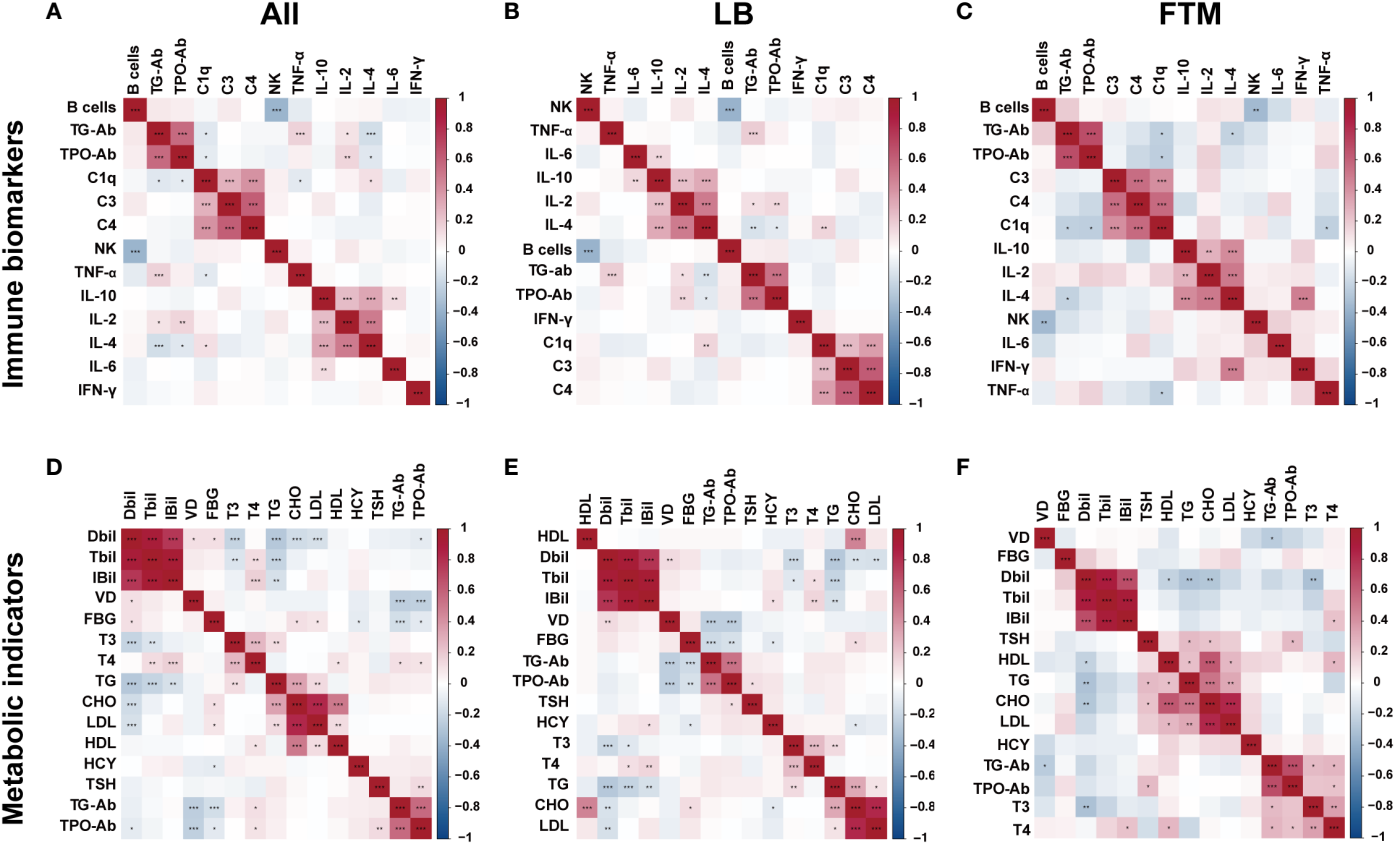
Heatmap of thyroid autoantibody-metabolite or thyroid autoantibody-immune biomarkers correlation and significance in all RSA patients, live birth RSA women (LB), and first-trimester miscarriage RSA women (FTM). The two-color heat map visually represents the association between thyroid autoantibody concentration and immune factors or metabolic factors, with red indicating positive correlation and blue indicating negative correlation. Asterisks denote significance: *p<0.05; **p<0.01; ***p< 0.001. **(A)** Heatmap of thyroid autoantibody-immune biomarkers correlation in all RSA women. **(B)** Heatmap of thyroid autoantibody-immune biomarkers correlation in live birth RSA women. **(C)** Heatmap of thyroid autoantibody-immune biomarkers correlation in first-trimester miscarriage RSA women. **(D)** Heatmap of thyroid autoantibody-metabolite correlation in all RSA women. **(E)** Heatmap of thyroid autoantibody-metabolite correlation in live birth RSA women. **(F)** Heatmap of thyroid autoantibody-metabolite correlation in miscarriage RSA women.

Based on a correlation analysis of immune biomarkers with TG-Ab or TPO-Ab, four showed significant correlations in all RSA patients, including C1q, TNF-α, IL-2, and IL-4. When this association was explored in groups of women with different pregnancy outcomes, **Figure 3** shows the correlation between TG-Ab or TPO-Ab and IL-2, IL-4 in the live-birth group, whereas in the first-trimester miscarriage group, TG-Ab, or TPO-Ab was associated with C1q, IL-2. Immune and metabolic factors consistently associated with TPO-Ab and TG-Ab in RSA patients were IL-4, Free T4, fasting glucose, and vitamin D (**Supplementary Figures 2**, **3**).

## 4 Discussion

This study investigated differences in metabolic markers, immune biomarkers, and pregnancy outcomes among RSA women with different thyroid autoimmune (TAI) status and risk factors for early pregnancy loss in TAI-positive RSA patients. Results showed that TSH levels significantly differed in RSA patients with and without TAI. Previous studies have shown that an increased risk of poor maternal and fetal outcomes may occur in pregnant women with abnormal thyroid hormone secretion (6–8), and our results also show that the rate of first-trimester miscarriages was higher in TAI-positive women with a borderline statistical difference compared to TAI-negative women. In this study, the live birth rate after 28 weeks was lower in the TAI-positive group, and the early miscarriage rate was higher in the positive group. Furthermore, when stratified by clinical characteristics, the proportion of women with live births was lower in TAI-positive women under certain clinical features such as older age, normal BMI, normotensives, previous live births, and more previous miscarriages. The HCY was the only metabolic indicator found to be different between women with first-trimester miscarriage and women with live births. At the same time, there was no significant difference in the immune biomarkers between RSA women with two pregnancy outcomes. TAI was another factor that significantly differed between the two groups.

When categorized according to TAI, it was observed that specific immune biomarkers exhibited variations between the two pregnancy outcomes of RSA patients. Complement c4 was identified as a risk factor for miscarriage in TAI-negative RSA women, whereas IL-6 was a protective factor for the same in RSA women with TAI. A case-control study supported elevated C4d at the maternal-fetal interface in women with unexplained recurrent miscarriages, which may reflect that these women have abnormal immunity against the fetus (15). Kwak. J. et al. previously found that cytokine imbalance may be essential in RSA patients (16). Cytokines are crucial in establishing a tolerogenic environment at the maternal-fetal interface for the semi-allogeneic fetus, particularly in the shift from a Th-1 to a Th-2 cytokine profile. Compared to women who test negative for thyroid antibodies, women with autoimmune thyroid disease and reduced fertility typically exhibit elevated levels of pro-inflammatory immune cytokines from Th-1 cells (such as IFN-γ), along with decreased levels of IL-4 and IL-10 produced by Th-2 immune cells (17). In this study, it was observed that IL-6 levels were elevated in live-birth women with recurrent spontaneous abortions who had thyroid autoimmune. This indicates that Th-2 cytokines may have a significant role in a successful pregnancy, whereas an excessive activation of pro-inflammatory Th-1 cells may impede a successful pregnancy.

This study found that the TAI positivity rate was 18.6% among recurrent spontaneous abortion patients, consistent with prior research findings. In comparison, the prevalence of TAI in ordinary women of childbearing age ranges from 8% to 14% (18, 19). Thyroid peroxidase (TPO) is a critical enzyme in the biosynthesis of thyroid hormones and is located on the apical surface of thyroid follicular cells (20). Anti-TPO antibodies (TPO-Ab) can be found in as many as 25% of the general population, with a greater prevalence among females and an increase with age (21). In specific TPO-Ab-positive individuals, particularly during pregnancy, adequate intrathyroidal hormone secretion may take place despite serum thyroid hormone levels falling within the normal range (22), potentially resulting in adverse pregnancy outcomes (23, 24). We screened important factors for adverse outcomes in women with TAI-positive RSA and constructed a risk model to predict their risk of adverse pregnancy outcomes. In our study, age and interleukin-6 demonstrated significant associations with pregnancy outcomes in women afflicted with TAI-positive recurrent spontaneous abortion (RSA). These two variables were among the seven factors integrated into the formulated risk model. In addition, metabolic and immune indicators are among the predictors of the established prediction risk model. Successful pregnancy and live birth might require a unique and complex immune status and an appropriate overall metabolic state. Kwak et al. reported a notable elevation in the proportion of CD19+ B cells in RSA pregnant women compared to normal women (16). Later, Jabłonowska et al. obtained similar results, demonstrating analogous outcomes by observing an increased rate and levels of peripheral B cells in early pregnancy RSA patients (25). After implantation, the female immune system induces tolerance to the embryo, but tolerance induction is incomplete in an overactive immune system.

This study revealed a correlation between TPO-Ab concentration and levels of vitamin D, blood glucose, IL-4, and free T4. Thyroid peroxidase (TPO) is a critical enzyme in the biosynthesis of thyroid hormones and is located on the apical surface of thyroid follicular cells (20). Potential mechanisms for TPO-Ab-induced pregnancy complications include: 1) the presence of circulating TPO-Ab may signify subclinical dysfunction; 2) direct impact of TPO-Ab on the placenta; 3) its role as a marker of general immune imbalance, influencing the immune relationship between the mother and the fetus (26). In parallel investigations scrutinizing immune mechanisms, the presence of thyroid autoimmunity as a predictor for systemic autoimmune disorders demonstrates close association with repeated implantation failure, early pregnancy loss, and adverse pregnancy outcomes (3, 27). Some studies suggest that thyroid antibodies may not exert direct effects on embryo quality but could contribute to a diminished clinical pregnancy rate, potentially attributed to compromised maternal immune regulation (28). Regarding the impact of TAI on pregnancy outcomes, divergent findings have emerged in the literature. Some studies have reported no discernible association between the presence of TPO-Ab and rates of miscarriage or successful deliveries (29, 30). In contrast, others reported higher rates of miscarriage and lower delivery rates in TPO-Ab-positive patients compared to negative patients due to shifts in metabolic and immune status (31–33). Vitamin D deficiency is considered a trigger for autoimmune diseases and is decreased in patients with autoimmune thyroid diseases. Vitamin D plays a pivotal role in regulating antigen-presenting cells such as dendritic cells, monocytes, and macrophages, as well as T cells and B cells, thereby exerting a crucial influence on immune function (34). Research has established a correlation between lower levels of vitamin D and the onset of autoimmune conditions such as autoimmune thyroiditis (35, 36).

This study first discussed the differences in metabolic markers and immune biomarkers among RSA women with different TAI status, correlating these differences with various pregnancy outcomes. The investigation unveiled that that metabolic characteristics and immune biomarkers serve as potential predictive indicators for discerning pregnancy outcomes in RSA women, thereby significantly augmenting our comprehension of the interplay between metabolism, immune status, and pregnancy outcomes within this demographic. We acknowledge the limitation of the current study that was conducted as a single center study. The relatively modest sample size and the exclusive focus on a single-center retrospective design necessitate caution in extrapolating findings universally. Potential biases stemming from allocation, selection, geographical confinement, and recall limitations might have influenced the study outcomes. The absence of comprehensive treatment information precluded an in-depth analysis of treatment effects on the research findings, potentially impacting the observed results. Future investigations should strive to mitigate these limitations by conducting expansive, multicenter, prospective randomized trials to establish robust, high-quality evidence. This endeavor will ensure a more comprehensive understanding of the intricate relationship between metabolic, immunological factors, TAI status, and pregnancy outcomes in RSA women, facilitating improved clinical management and informed decision-making. In conclusion, the prevalence of TAI in women with RSA is 18.6%. TAI-positive RSA patients have a higher first-trimester miscarriage rate and lower live birth rate, which may be related to metabolic and immune shifts in TAI-positive RSA patients.

## Data availability statement

The original contributions presented in the study are included in the article/**Supplementary Material**. Further inquiries can be directed to the corresponding authors.

## Ethics statement

The studies involving humans were approved by The Ethics Committee of The Third Affiliated Hospital of Wenzhou Medical University. The studies were conducted in accordance with the local legislation and institutional requirements. The participants provided their written informed consent to participate in this study.

## Author contributions

JZ: Data curation, Formal analysis, Investigation, Resources, Software, Writing – original draft. ZS: Data curation, Writing – review & editing. HY: Conceptualization, Formal analysis, Methodology, Software, Visualization, Writing – review & editing. ZC: Conceptualization, Writing – review & editing, Funding acquisition, Project administration, Supervision.
