# Supplementary material for: The effects of metabolic indicators and immune biomarkers on pregnancy outcomes in women with recurrent spontaneous abortion: a retrospective study
Source: Front Endocrinol (Lausanne). 2024 Jan 17;14:1297902. doi: 10.3389/fendo.2023.1297902 (PMC10827934; doi:10.3389/fendo.2023.1297902)
Supplement: Supplementary file 1 [file DataSheet_1.pdf]

Supplementary Material

1 Supplementary Data

Abbreviations: FTM, first-trimester miscarriage; LB, live birth; TAI, thyroid autoimmunity; BMI**,** body mass index; TSH, thyroid stimulant hormone; T3**,** triiodothyronine; T4, thyroxine**;** HCY**,** thyroid stimulant hormone; VD, 25 hydroxyvitamin D; FBG, fasting blood glucose ; CHO, cholesterol; TG, triglyceride; HDL-c, high-density lipoprotein cholesterol ; LDL-c, low-density lipoprotein cholesterol; TBil, total bilirubin; DBil, direct bilirubin; IBil, indirect bilirubin; NK: natural killer; IL, interleukin; TNF, tumor necrosis factor; IFN, interferon

2 Supplementary Figures and Tables

2.1 Supplementary Tables

Supplementary Table 1. Comparison of pregnancy outcomes between women with and without TAI.

Variables	TAI^- N = 376[1]	TAI^+ N = 85[1]	p-value[2]
**Pregnancy outcomes**			0.061
LB	314 (83.5%)	63 (74.1%)	
FTM	62 (16.5%)	22 (25.9%)	

Supplementary Table 2. Comparison of immune biomarkers and metabolic indicators levels between women with and without TAI.

Variables	TAI^- N = 376[1]	TAI^+ N = 85[1]	p-value[2]
HCY (umol/L)	6.10 (5.30, 7.00)	6.30 (5.70, 7.30)	0.029
**VD (ng/ml)**	21 (16, 26)	14 (11, 17)	**<0.001**
FBG (mmol/L)	4.61 (4.18, 5.15)	4.25 (3.89, 4.74)	<0.001
CHO (mmol/L)	3.87 (3.46, 4.37)	3.80 (3.32, 4.53)	0.759
TG (mmol/L)	0.90 (0.68, 1.30)	0.89 (0.69, 1.35)	0.743
HDL-c (mmol/L)	1.25 (1.09, 1.43)	1.30 (1.17, 1.45)	0.279
LDL-c (mmol/L)	2.17 (1.79, 2.60)	2.09 (1.71, 2.69)	0.780
Tbil (umol/L)	10.6 (8.1, 14.2)	10.8 (8.2, 13.3)	0.948
Dbil (umol/L)	4.20 (3.20, 5.60)	4.10 (2.80, 5.40)	0.203

(*Continued*)

Supplementary Table 2 | Continued

IBil (umol/L)	6.4 (4.6, 8.7)	6.7 (4.8, 8.4)	0.398
Complement C3 (g/l)	0.90 (0.79, 1.05)	0.92 (0.76, 1.08)	0.582
Complement C4 (g/l)	0.20 (0.16, 0.25)	0.18 (0.15, 0.24)	0.231
Complement C1q (g/l)	172 (150, 192)	163 (142, 184)	0.050
NK (%)	10 (7, 15)	9 (6, 14)	0.328
**Total B cells (%)**	**13.8 (10.6, 16.8)**	**14.9 (11.7, 18.3)**	**0.029**
**IL-2 (pg/ml)**	**0.72 (0.33, 1.18)**	**0.82 (0.50, 1.58)**	**0.004**
**IL-4 (pg/ml)**	**0.85 (0.44, 1.42)**	**0.68 (0.24, 1.06)**	**0.004**
**IL-6 (pg/ml)**	**2.15 (1.30, 3.29)**	**2.62 (1.69, 3.70)**	**0.008**
IL-10 (pg/ml)	0.94 (0.50, 1.52)	1.04 (0.54, 1.51)	0.740
IFN-γ (pg/ml)	0.88 (0.50, 1.23)	0.94 (0.50, 1.52)	0.192
TNF-α (pg/ml)	1 (0, 2)	1 (1, 3)	0.109

[1] *Median (IQR); n (%)*

[2] *Wilcoxon rank sum test; Pearson's Chi-squared test*

2.2 Supplementary Figures

Supplementary Figure 1. Important factors related to pregnancy outcome screened by random forest in TAI-positive RSA patients.

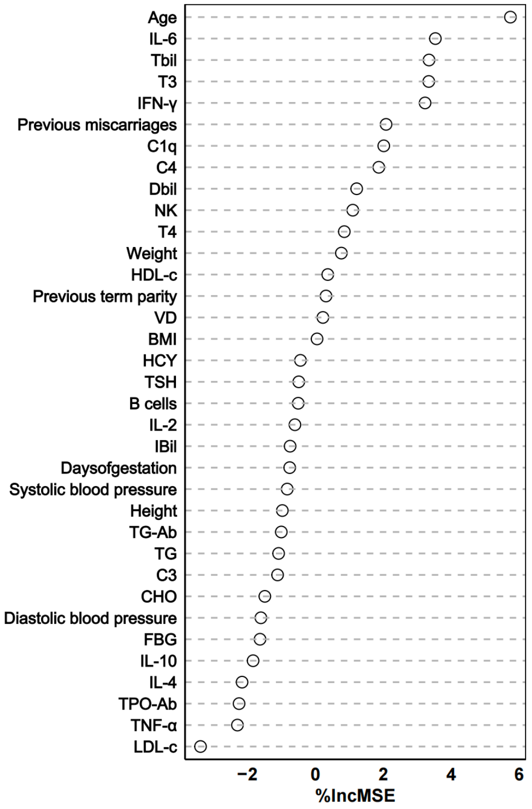

Supplementary Figure 2. Metabolic or immune indicators related to TG-Ab in RSA patients. Linear regression shows correlations (black lines) and peak plots on the x- and y-axes indicate the distribution of the factors they represent.

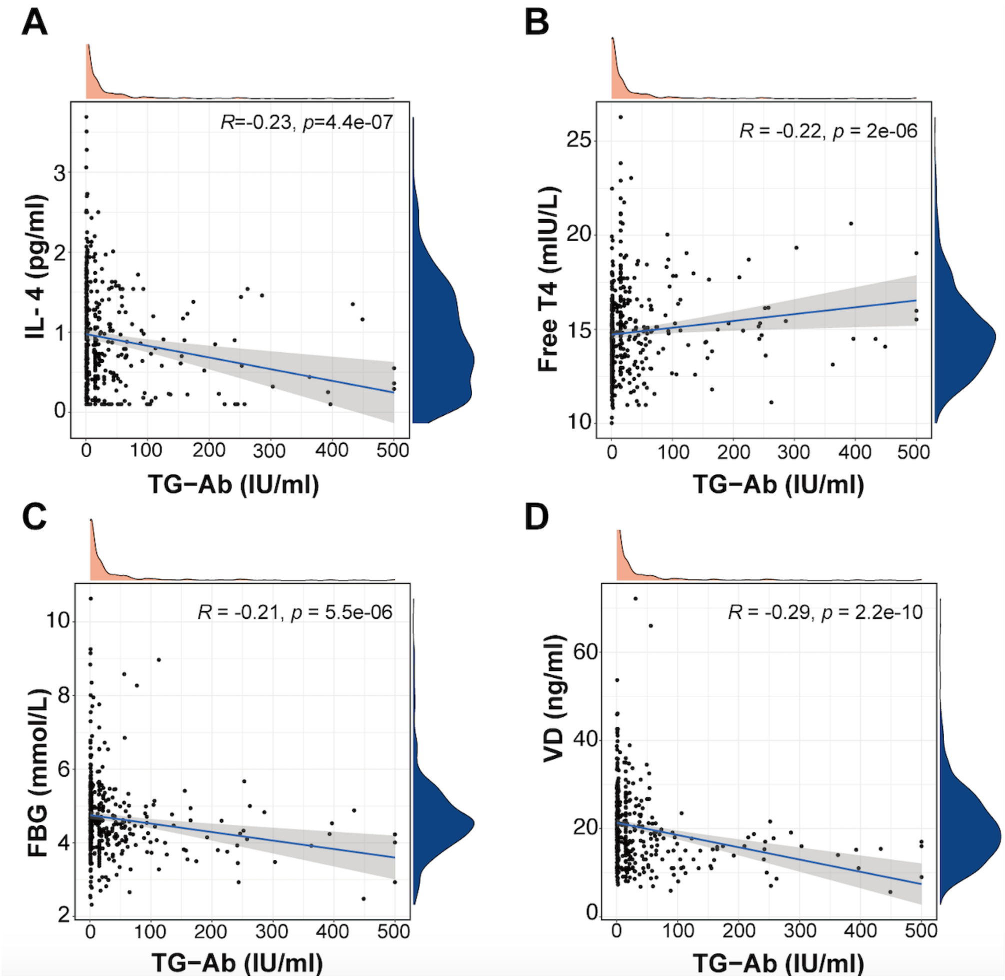

A. Negative correlation between TG-Ab and IL-4 (R<-0.2, p<0.05)

B. Positive correlation between TG-Ab and Free T4 (R>0.2, p<0.05)

C. Negative correlation between TG-Ab and FBG (R<-0.2, p<0.05)

D. Negative correlation between TG-Ab and VD (R<-0.2, p<0.05)

Supplementary Figure 3. Metabolic or immune indicators related to TPO-Ab in RSA patients. Linear regression shows correlations (black lines) and peak plots on the x- and y-axes indicate the distribution of the factors they represent.

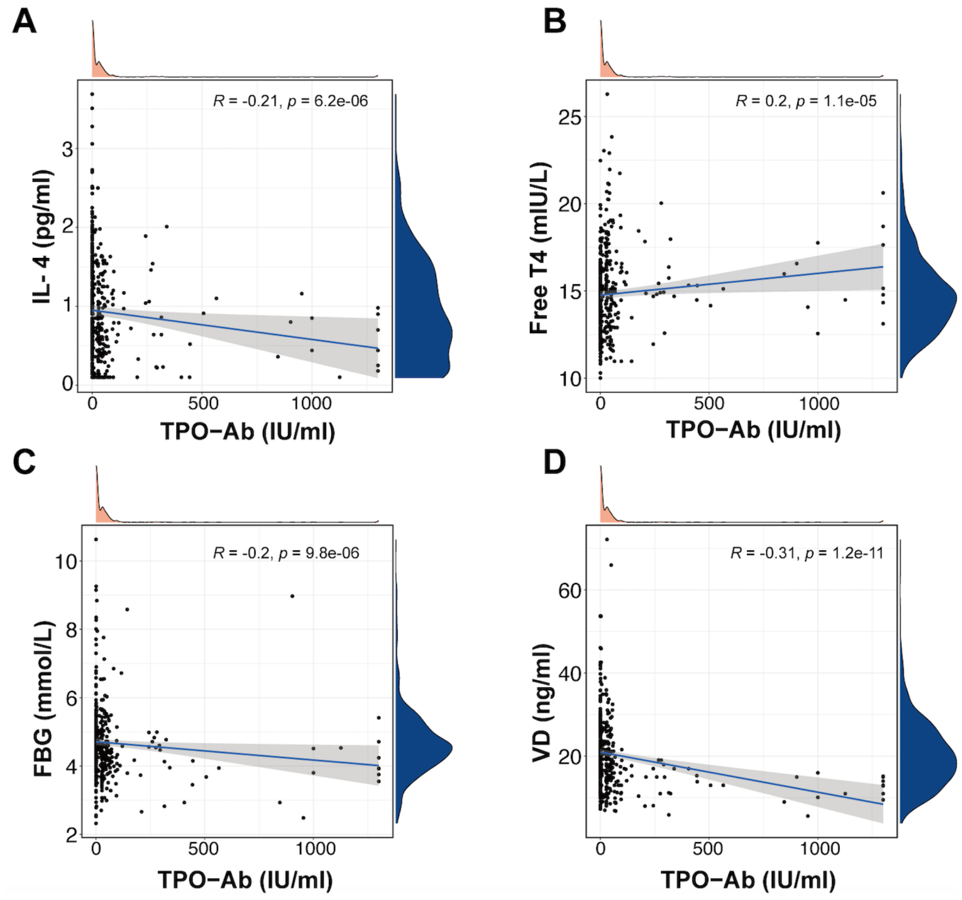

A. Negative correlation between TPO-Ab and IL-4 ($R<-0.2$, $p<0.05$)

B. Positive correlation between TPO-Ab and Free T4 ($R>0.2$, $p<0.05$)

C. Negative correlation between TPO-Ab and FBG ($R<-0.2$, $p<0.05$)

D. Negative correlation between TPO-Ab and VD ($R<-0.2$, $p<0.05$)
